# How to undertake a research project and write a scientific paper

**DOI:** 10.1308/10.1308/003588412X13171221590331

**Published:** 2012-07

**Authors:** M Rhodes

**Affiliations:** University of East Anglia,UK

**Keywords:** Research Techniques, Research Activities, Publications, Journal Article

## Abstract

Research and publishing are essential aspects of lifelong learning in a surgical career. Many surgeons, especially those in training, ask for guidance on how they might start a simple project that may lead to a publication. This short paper offers some practical guidelines on the subject.

## How to get started with a project

How to get started varies depending on whether the project is suggested by a trainer or educational supervisor. Projects suggested by a senior are always offered as an encouragement to a trainee, who should be careful not to respond in a negative way by ignoring the suggestion, coming up with a string of excuses or doing the project badly! Here are some simple steps that may contribute to an organised start on the project. You need a protocol but first you must be clear about what the project will involve.

1. Undertake a literature search on the suggested topic.
2. Read all the papers from the last ten years and summarise them on a single page of A4.
3. Make a note of how many similar series have been produced, their size, the length of follow-up and any special aspects of the subject that have already been addressed.
4. List aspects of the topic that have not been well covered, perhaps morbidity or surgery for rare indications, or long-term outcomes.
5. Discuss your thoughts on the subject with your colleagues.
6. With the strengths and weaknesses of the current literature clear in your own mind, summarise your thoughts in bullet points on a single side of A4 and arrange ten minutes to discuss them with the senior who suggested the topic.

The six steps listed above can be easily completed within a couple of weeks. Once you have discussed and agreed the aims of the project as well as how they can be achieved, you can write your protocol. It is also possible that having studied the literature you decide the suggested project is unlikely to add to our current knowledge and that another topic might be better studied.

A protocol and approval from your trust’s research and development (R&D) department as well as from the research ethics committee (REC) are needed before you begin a research project. If you are planning a service evaluation, REC approval may not be needed. When you have secured the approvals, the process of collecting the data begins.

Examining a case series, there may be hundreds of medical records that need to be studied and it is crucial to draw up a ‘proforma’ on which to record patient data. This should ideally fill no more than one or two sides of A4 and needs to include all the data that you have decided to collect for your particular study. It is crucial not to leave out a dataset you might later wish to look at but on the other hand it is also important not to collect too many data. Because of this fine balance, it is important to draw up a proforma and agree its composition with your supervisor and any co-workers on the project *before* starting to collect data from the medical records.

Data collection can be time consuming and it may be that several colleagues can work on this to speed the project along. Once all the data proformas are filled in, the data need to be entered into the database, spreadsheet or statistical package of your choice. It is best to use the software favoured by the department or colleagues in medical statistics.

Having looked at the data, discipline yourself to produce a succinct summary on one side of A4. Again, arrange a meeting with your supervisor and any other co-workers to discuss the findings, and give everyone the opportunity to comment and correct the summary. Once the findings are agreed, you are ready to write up the project.

## Self-generated projects

Sometimes you will want to develop an idea of your own. It is even more important with a self-generated project****to do a thorough literature search to make sure that your ideas will contribute to our knowledge. The discussion of a more ambitious project like a randomised trial should be with as many colleagues as possible, both for advice and also to garner support for your idea. Having produced a single side of A4 summarising your idea, identify a senior colleague who can advise you and proceed as described above.

As noted previously, REC approval is needed for any clinical research involving patients or their data. You will need to prepare an application on the Integrated Research Application System website (https://www.myresearchproject.org.uk/). If you have never done this before, seek advice from your trust’s R&D department. REC approval is time consuming; the following comments may help:

1. Much of your initial work producing a summary of your idea will be helpful in completing the ethics committee form. It is crucial that submission to your local ethics committee is checked by all your co-workers.
2. Colleagues from medical statistics and any other parallel disciplines such as radiology or medical chemistry need to be involved right at the start of this formal submission so that all aspects of the study are academically correct. It is especially important to have expert statistical input because it is very demoralising to finish a trial only to be told that your study is woefully underpowered and cannot answer the question that it set out to address!
3. It is wise to present your idea to the committee in person as this can save time and iron out minor misunderstandings. These ‘glitches’ in an ethics submission can soak up months of precious time and a personal meeting with the REC can help to avoid them.
4. Many institutions also have research governance or internal review boards that must also pass a project after it has gained ethical approval. Their role is often to assess the financial and organisational impact of a study.

This process seldom takes less than 3 months and may take nearly 12 months. Do not be disheartened by this. If your study is worth doing, then it is worth persevering.

The recording of data using a concise proforma, entry into appropriate computer software and production of a summary of your findings are all conducted in the same way as in the first section of these guidelines.

## Writing up a study

One of the most challenging aspects of surgical research is writing a paper. Putting together a manuscript for submission to a journal can be broken down into several simple and relatively self-contained steps:

1. *Journal guidelines*: All journals have a set of instructions for potential authors. The suggestions below are an overall guide to writing a paper but should be viewed in the context of the specific guidelines on submission to the journal you have chosen for your work.
2. *Title*: Keep this simple and concise.
3. *Authorship*: This topic may be a source of some problems. My own observation about authorship is that if you leave somebody out who feels they have contributed to your project, you can make an enemy for life! It is easy to forget colleagues, especially when a project has run for several years. Try, within the internationally agreed authorship guidelines, to include all colleagues who have contributed significantly to your study.

The order of authorship may also cause problems. It is generally agreed that the main researcher who also produced the first draft of the paper is the first author. The second author has usually been the second main contributor to the project. The last author is the senior person supervising the work. Between these positions come all other authors who fulfil the guidelines for authorship. If in any doubt about who should or should not be in the authorship, discuss it with your senior author.

All papers have a corresponding author responsible for answering queries after submission of the manuscript. It is best if he or she is a permanent member of the department as queries may arrive several years after a paper is published.

1. *Abstract*: This is usually 200–250 words and should be written in the style of the journal. Generally, this includes sections on background, methods, results and conclusions.
2. *Introduction*: This should introduce the reader to the subject covered in the study and explain why this particular study has been undertaken. It should be kept to two or three paragraphs. The first paragraph sets the scene and summarises the current literature. The second paragraph should justify why this particular study or series of cases has been put together.
3. *Patients and methods*: The most frequent mistake in this section is to include results as well as patient details. It is important to stick to describing the study population, how they were collected and, crucially, how any analyses were undertaken. Always describe what statistical tests were used and justify why they were appropriate.
4. *Results*: These should be presented concisely with as few tables or figures as possible. Use a logical sequence and follow the same sequence in the methods and discussion sections.
5. *Discussion*: Many surgeons don’t know where to start in this section! Over 25 years I have found the following outline helps to clarify one’s thoughts when discussing a study. Using these five headings can keep the discussion concise, relevant and, most importantly, just five paragraphs in length!
  - *What are the main findings of your work?* State clearly what you can conclude from your observations, taking care not to overestimate what you can conclude.
  - *Why are these findings valid (sample size, methods etc)?* Explain what leads you to conclude that your findings may be relied on. Also make sure you highlight any potential weaknesses in your data and consider other potential confounding variables that might invalidate your conclusions.
  - How do your observations compare with other work in the same area? Discuss how results from your work compare with other papers on the same subject, either explaining similarities or examining differences.
  - *Any other business?* Are there any unexpected side observations that merit separate discussion? This might include unexpected complications in a trial or a unique subset of patients in a clinical series.
  - Restate your main findings and suggest what further work might be helpful in providing more information on the topic of your project.
6. *References*: Make sure these are presented in the style of the journal you have selected.

## Publication of the paper

This can be the biggest hurdle you have to clear! Some basic rules will help to make this easier. First, never submit a paper without all authors having read it and agreed to the content. Second, never submit a paper to more than one journal at a time. Finally, remember that submission is not the end of your paper but just the beginning.

Selection of the right journal is important. On the basis of their impact factor, journals may be divided into four divisions. Think of it like the football league! The premier division contains journals with impact factors greater than 10, the second division those with impact factors from 5 to 10, the third division with impact factors from 1 to 5 and, finally, the fourth division with impact factors less than 1. Just as with football, journals may be promoted or relegated so it is wise to check online for a journal’s current impact factor.

Discuss with your co-workers what your target journal should be. It is acceptable to aim just higher than you think your paper ranks but obviously pointless sending a small case series to one of the premiership journals. A second consideration is which articles have appeared in your target journal over the last 12 months. If there have been one or more papers on the same subject as your work, it may be better to select an equally ranked journal that has not had a paper on your topic for several years.

Peer review is the process used by journals to select papers for publication. Many papers are rejected immediately but those deemed of potential interest are sent out for peer review. This process usually takes 3–4 months (although some journals such as the *Annals of The Royal College of Surgeons of England* have a much quicker turnaround). There are four potential outcomes:

1. Accept without corrections – this is very rare!
2. Minor corrections needed followed by resubmission for publication
3. Major corrections needed and resubmission invited but without any promise to publish
4. Major criticisms and rejection (for most major journals this is the single largest category of outcomes)

When you receive the reviewer’s comments don’t take them personally! The best way to regard the reviewer’s criticisms is as helpful suggestions to improve your paper. It is crucial to deal with each of the reviewer’s comments carefully, systematically and politely. If possible, respond to the comments within a few days of receiving them.

If your paper has been rejected, then the reviewer’s comments are an excellent set of suggestions to improve the manuscript for submission to another journal. This should probably be in one division lower than your first submission. Again, there is no reason to delay resubmission to another journal more than a few days. Make sure that all possible advice on rewriting and correcting your paper is taken and your work will almost certainly get published eventually!

